# Device-independent quantum key distribution with random key basis

**DOI:** 10.1038/s41467-021-23147-3

**Published:** 2021-05-17

**Authors:** René Schwonnek, Koon Tong Goh, Ignatius W. Primaatmaja, Ernest Y.-Z. Tan, Ramona Wolf, Valerio Scarani, Charles C.-W. Lim

**Affiliations:** 1 Department of Electrical & Computer Engineering, National University of Singapore, Singapore, Singapore; 2 Centre for Quantum Technologies, National University of Singapore, Singapore, Singapore; 3 Institute for Theoretical Physics, ETH Zürich, Zürich, Switzerland; 4 Institut für Theoretische Physik, Leibniz Universität Hannover, Hannover, Germany; 5 Department of Physics, National University of Singapore, Singapore, Singapore

**Keywords:** Quantum information, Theoretical physics

## Abstract

Device-independent quantum key distribution (DIQKD) is the art of using untrusted devices to distribute secret keys in an insecure network. It thus represents the ultimate form of cryptography, offering not only information-theoretic security against channel attacks, but also against attacks exploiting implementation loopholes. In recent years, much progress has been made towards realising the first DIQKD experiments, but current proposals are just out of reach of today’s loophole-free Bell experiments. Here, we significantly narrow the gap between the theory and practice of DIQKD with a simple variant of the original protocol based on the celebrated Clauser-Horne-Shimony-Holt (CHSH) Bell inequality. By using two randomly chosen key generating bases instead of one, we show that our protocol significantly improves over the original DIQKD protocol, enabling positive keys in the high noise regime for the first time. We also compute the finite-key security of the protocol for general attacks, showing that approximately 10^8^–10^10^ measurement rounds are needed to achieve positive rates using state-of-the-art experimental parameters. Our proposed DIQKD protocol thus represents a highly promising path towards the first realisation of DIQKD in practice.

## Abstract (web summary)

Device-independent quantum key distribution aims at the ultimate quantum-based unconditional security, but current protocols’ rates are quite far from anything practical. The authors’ protocol narrows this gap by using two randomly chosen key generating bases instead of one.

## Introduction

**Fig. 1:**
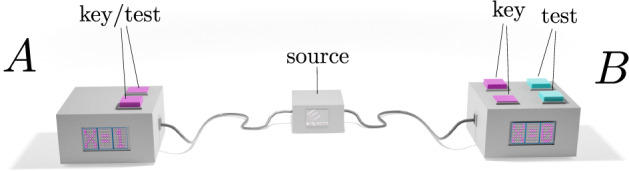
Robust DIQKD. Alice and Bob use uncharacterised devices to perform measurements on a quantum state that is created by a source that is potentially controlled by an adversary (Eve). In the proposed protocol, Alice has two possible inputs (measurement settings, magenta buttons) which are used for key generation and for running the CHSH Bell test, and Bob has four possible inputs grouped into two sets (magenta/cyan buttons): the magenta buttons are used for key generation while the cyan buttons are used for running the CHSH Bell test.

The basic task of DIQKD^1–5^ is to distribute a pair of identical secret keys between two users, called Alice and Bob, who are embedded in an untrusted network. To help them in their task, Alice and Bob are each given a measurement device, which they use to perform random measurements on a sequence of entangled systems provided by an adversary called Eve (see Fig. 1). The main advantage of DIQKD is that the measurement devices need not be characterised—Alice and Bob only need to verify that the input–output statistics of the devices violate a CHSH Bell inequality^6,7^. As such, DIQKD represents the pinnacle of cryptography in terms of the number of assumptions required. More specifically, it only asks that (1) the users each hold a trusted source of local randomness, (2) their laboratories are well isolated, (3) they use trusted algorithms for processing their measurement data, (4) if the devices are reused for multiple instances of the protocol, the outputs in later instances do not leak information about earlier outputs, (5) they possess sufficient pre-shared keys to implement information-theoretically secure authenticated (public) channels, and that (6) quantum theory is correct. Given these basic assumptions (which are, in fact, standard assumptions in cryptography), one can then show that DIQKD is information-theoretically secure^8–10^. We note that assumption (4) is needed to address issues with protocol composition^11^ and memory attacks^12^, because information-theoretic security may be violated if the protocol’s public communication leaks some information about the private data from earlier instances.

The practical implementation of DIQKD, however, remains a major scientific challenge. This is mainly due to the need to have extremely good channel parameters (i.e. high Bell violation and low bit error rate), which in practice requires ultra-low-noise setups with very high detection efficiencies; though in recent years the gap between the theory and practice has been significantly reduced owing to more powerful proof techniques ^9,10,13^ and the demonstrations of loophole-free Bell experiments^14–17^. The present gap is best illustrated by Murta et al.^18^, whose feasibility study showed that current loophole-free Bell experiments are just short of generating positive key rates assuming the original DIQKD protocol^2,3^ (see the dashed line in Fig. 3).

To improve the robustness of DIQKD, researchers have taken several approaches, from heralding-type solutions^19–21^, local precertification^22,23^, local Bell tests^24^, to two-way classical protocols^25^. However, none of these proposals are truly practical, for they are either more complex in implementation or provide only very little improvements in the channel parameters. Here, we show that a simple variant of the original DIQKD protocol is enough to obtain significant improvements in the channel parameters.

## Results and discussion

To start with, we note that in the original protocol introduced by Acín et al.^3^, the key generating basis is predetermined and known to Eve. For concreteness, let Alice’s and Bob’s measurement settings be denoted by *X* ∈ {0, 1} and *Y* ∈ {0, 1, 2}, respectively, and let the corresponding outcomes be denoted by *A*_*X*_ ∈ {0, 1} and *B*_*Y*_ ∈ {0, 1}. The secret key is derived from the events in which Alice and Bob choose *X* = 0 and *Y* = 0, respectively. The remaining measurement combinations are then used for determining the CHSH violation. Our DIQKD proposal is essentially the same as the original protocol, except that we introduce an additional measurement setting for Bob and now generate the secret key from both of Alice’s measurements. This additional setting is needed so that Bob has a measurement that is aligned with Alice’s additional key generating basis to obtain correlated outcomes (like in the case of the original protocol). Hence in our proposal, the key generation events are those where Alice and Bob choose *X* = *Y* = 0 and *X* = *Y* = 1. Below, we describe the proposal in detail (Box 1).

In the parameter estimation step of the protocol, note that when the inputs are not uniformly distributed i.e. *p* ≠ 1/2, the CHSH value is to be computed in terms of the conditional probabilities *P*(*A*_*X*_, *B*_*Y*_∣*X*, *Y*) rather than the unconditioned probabilities *P*(*A*_*X*_, *B*_*Y*_, *X*, *Y*) directly. We remark that this does not introduce a measurement-dependence^26^ security loophole, because the choice of inputs is still independent of the state.

It is well known that incompatible measurements are necessary for the violation of a Bell inequality and that such measurements are not jointly measurable and hence cannot admit a joint distribution^27–29^. The intuition behind our proposal roughly follows along this line and exploits two related facts: (1) the key generation measurements of Alice must be incompatible for *S* > 2 and (2) Eve has to guess the secret key from two randomly chosen incompatible measurements.

When the secret key is only generated from a single measurement, like in the original DIQKD protocol, Eve’s attacks are basically limited only by the observed CHSH violation and thus the monogamy of entanglement^30^. Eve, however, knows which measurement is used for key generation and hence can optimise her attack accordingly. On the other hand, if the secret key is generated from a random choice of two possible measurements, Eve faces an additional difficulty. Namely, in order to achieve a CHSH violation, the two measurements cannot be the same, and it is known^2^ that for CHSH-based protocols, different measurements can give Eve different amounts of side-information; note that this is not the case for BB84 and six-state QKD protocols. Therefore, at least one of the measurements will not be the one that maximises Eve’s side-information, giving an advantage over protocols based only on one key-generating measurement (Eve cannot tailor her attack to the measurement in each round individually, since she does not know beforehand which measurement will be chosen).

In the following, we first quantify the security of the protocol using the asymptotic secret key rate, *K*_*∞*_. This quantity is the ratio of the extractable secret key length to the total number of measurement rounds *N*, where *N* → *∞*. In the asymptotic limit, we may also take *q* → 1, which maximises the so-called sifting factor^31^ and get

1 $${K}_{\infty }={p}_{s}{r}_{\infty },$$

where *p*_*s*_: = *p*^2^ + (1 − *p*)^2^ is the probability of having matching key bases, and *r*_*∞*_ is the secret fraction^32^. The latter is given in terms of entropic quantities and reads

2 $${r}_{\infty }:=\;	\underbrace{\lambda H({A}_{0}{| }E)+(1-\lambda )H({A}_{1}{| }E)}_{\begin{array}{c}H(Z| E{{\Theta }})\end{array}}\\ 	-\lambda h({Q}_{{A}_{0}{B}_{0}})-(1-\lambda )h({Q}_{{A}_{1}{B}_{1}}),$$

where $h(x):=-x{\mathrm{log}}\,(x)-(1-x){\mathrm{log}}\,(1-x)$ is the binary entropy function, *λ*: = *p*^2^/*p*_*s*_, ${Q}_{{A}_{X}{B}_{Y}}:=P({A}_{X}\;\ne\; {B}_{Y}| X,Y)$ is the quantum bit error rate (QBER) for *X*, *Y*, and *E* is Eve’s quantum side-information gathered just before the error correction step. Here, *Z* = *A*_Θ_ and Θ ∈ {0, 1} is the random variable denoting Alice’s basis choice conditioned on the event either *X* = *Y* = 0 or *X* = *Y* = 1. Moreover, *E* refers to quantum side information possessed by Eve. Hence, Eve’s knowledge is fully described by *E*Θ. The second line in equation (2) is the amount of information leaked to Eve during the error correction step (decoding with side-information Θ).

The main challenge here is to put a reliable lower bound on the conditional von Neumann entropy *H*(*Z*∣*E*Θ), which measures the amount of uncertainty Eve has about *Z* given side-information *E*Θ, using solely the observed CHSH violation, *S*. To this end, we employ a family of device-independent entropic uncertainty relations^33,34^, which we can solve efficiently and reliably using a short sequence of numerical computations. More specifically, we seek to establish weighted entropic inequalities of the form

3 $$\lambda H({A}_{0}| E)+(1-\lambda )H({A}_{1}| E)\ge {C}^{* }(S),$$

where C*(*S*) is a function of the observed CHSH violation, *S*. A proof sketch is outlined in the ‘Methods’ section and the complete analysis is provided in the accompanied Supplementary Note 1.

A commonly used noise model for benchmarking the security performance of different QKD protocols is the depolarising channel model^2,3,32^. In this noise model, all QBERs are the same and related to the CHSH value *S* via

4 $${Q}_{{A}_{0}{B}_{0}}={Q}_{{A}_{1}{B}_{1}}=Q=\frac{1}{2}\left(1-\frac{S}{2\sqrt{2}}\right).$$

**Fig. 2:**
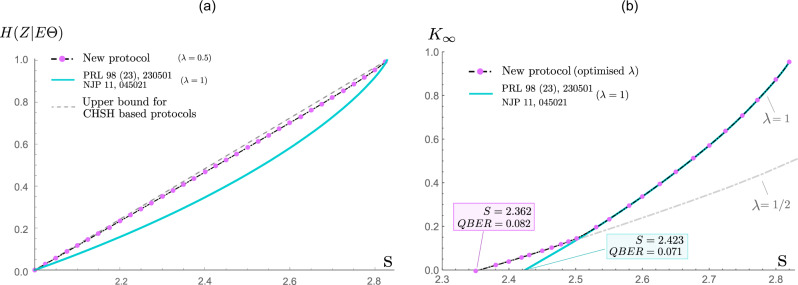
Secret key rate and uncertainty of Eve. **a** Assuming the validity of quantum theory and a given CHSH value *S* > 2, we show that our new protocol can establish and certify drastically more uncertainty *H*(*Z*∣*E*Θ) (close to the upper physical limit) than the best approach known before. **b** We consider a noise model (depolarising noise)^2,3^ that only depends on the CHSH value *S*. Now a larger amount of noise can be tolerated in order to establish a positive key rate *K*_*∞*_. In detail, we can decrease the critical CHSH value from 2.423 to 2.362. This corresponds to an increase of the critical bit-error-rate from 0.071 to 0.082, which brings a practical implementation of DIQKD into the reach of existing experiments.

Using this model, we compute the secret key rate and *H*(*Z*∣*E*Θ), which are presented in Fig. 2. Here, *λ* is a free parameter (i.e. a protocol parameter) that can be optimised by Alice and Bob (i.e. they optimise *p* = *P*(*X* = 0)) for a given pair of (*S*, *Q*). The result of this optimisation is remarkably simple: it is optimal to use a protocol with *λ* = 1/2 (uniformly random key generation bases) if *S* ⪅ 2.5(high noise) and set *λ* = 1, i.e. a fixed key generation basis otherwise (low noise). Surprisingly, there is no need to consider the intermediate values of 1/2 < *λ* < 1. In the case of the latter, our proposal reverts back to that of Acín et al.^1–3^ and the computed secret key rate appears to exactly match their analytical key rate bound. When using *H*(*Z*∣*E*Θ) as a performance metric (which only depends on *S* and thus applies to a general class of channel models satisfying this constraint), we observe that the uncertainty of Eve for our proposal is always higher than that of the original protocol for all $S\in (2,2\sqrt{2})$, see the right side of Fig. 2. In fact, for $\lambda =\frac{1}{2}$ our proposal is nearly optimal, in the sense that the bound on *H*(*Z*∣*E*Θ) is very close to the linear bound, which is the fundamental upper limit of Eve’s uncertainty given a fixed *S*. However, while it is optimal in this sense, choosing $\lambda =\frac{1}{2}$ is not always optimal in terms of producing the highest secret key rates, because the key rate is penalized by the sifting factor. Hence, *λ* = 1 is preferred for the region *S* ⪆ 2.5.

**Table 1:** Asymptotic rates for existing experiments (data from Murta et al.^18^, Table 4).

Label	Experiment	Year	Rate [bit/channel use]
(1)	Matsukevich et al.^64^	Cold atom 2008	0.004
(2)	Pironio et al.^65^	Cold atom 2010	0.118
(3)	Giustina et al.^66^	Photonic 2013	0
(4)	Christensen et al.^67^	Photonic 2013	0
(5)	Giustina et al.^15^	Photonic 2015	0
(6)	Shalm et al.^16^	Photonic 2015	0
(7)	Hensen et al.^14^	NV-centre 2015	0.057
(8)	Rosenfeld et al.^17^	Cold atom 2017	0.019
(9)	Liu et al.^68^	Photonic 2018	0
(10)	Liu et al.^69^	Photonic 2019	0
(11)	Li et al.^70^	Photonic 2019	0

Non-photonic experiments ((1, 2) and (7, 8)) now promise a positive keyrate. However, note that the experiments (1, 2) were performed in a single lab and therefore did not close the locality loophole. The photonic experiments (3, 4) also did not close the locality loophole. The more recent experiments (5, 6, 7, 8, 9, 10, 11) closed both locality and detection loopholes. Note that the value of QBER for experiment (8) provided by Murta et al.^18^ is based on the experiment of Henkel et al.^63^ while the QBER achievable by experiment (8) is estimated to be higher by the authors of experiment (8)^17^.

**Fig. 3:**
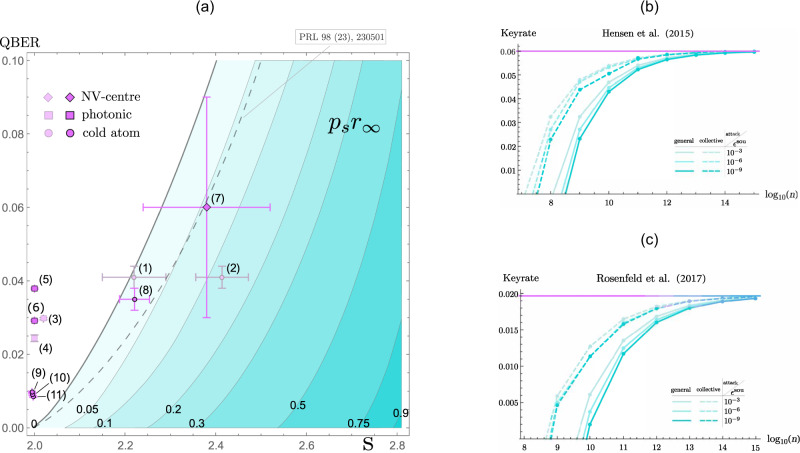
Rates for existing experiments. The contour plot (**a**) illustrates the asymptotic key rate *K*_*∞*_ = *p*_*s*_*r*_*∞*_ as a function of *S* and *QBER*. We marked the location of recent experiments (see Table 1). Our DIQKD proposal suggests that now a positive asymptotic key rate of reasonable magnitude is possible for experiments (7, 8). The plots **b**, **c** show the finite-size key rates as a function of number of rounds, for the choice *p* = 1/2 (which appears optimal at these noise levels). These plots are for the estimated parameters in Murta et al.^18^ for the Bell tests in (**b**) Hensen et al.^14^ and (**c**) Rosenfeld et al.^17^, respectively. The solid curves show the results for general attacks, while the dashed curves show the results under the assumption of collective attacks. The different colours correspond to different soundness parameters *ϵ*^sou^ (informally, a measure of how insecure the key is; see Supplementary Note 4) as listed in the inset legends, while the completeness parameter (the probability that the honest devices abort) is *ϵ*^com^ = 10^−2^ in all cases. The horizontal line denotes the asymptotic key rate. Note that only experiments (5, 6, 7, 8, 9, 10, 11) are loophole-free Bell tests, closing both detection and locality loopholes. On the other hand, experiments (1, 2, 3, 4) did not close the locality loophole.

In order to evaluate the feasibility of our proposal, we look at the existing list of loophole-free CHSH experiments^18^ and compute the corresponding secret key rates. Generally speaking, there are two types of Bell experiments: one based on measuring entangled photon pairs using high efficiency single-photon detectors, and the other based on event-ready systems^35^ using entanglement swapping between entangled photon pairs and atoms/NV-centres. Following along the lines of Murta et al.^18^, we prepare a feasibility region plot for the list of CHSH experiment therein, which is presented in Fig. 3. The immediate observation is that our DIQKD proposal significantly expands the region of channel parameters that give rise to positive key rates, thus substantially improving the robustness of DIQKD. The next observation is that event-ready loophole-free CHSH experiments^14,17^ are now well within the positive key region; as opposed to the original protocol where they are either in the insecure region or around the boundary. Unfortunately, CHSH experiments based on entangled photon pairs are still in the insecure region (also see Supplementary Note 2), although it should be mentioned that this observation holds only for our proposal and the original DIQKD protocol (Table 1).

Our results hence show that positive asymptotic key rates can be achieved by recent event-ready loophole-free experiments. This significantly improves over the original protocol^2^, which does not achieve positive key rates for any such experiments, even in the asymptotic limit (though Murta et al.^18^ describes prospective future improvements to NV-centre implementations, which may allow positive asymptotic rates). However, there are still a few experimental challenges. For one, we note that the event-ready CHSH experiments are fairly slow compared to their photonic counterparts; e.g. the event-ready experiment by Hensen et al.^14^ performed only 245 rounds of measurement during a total collection time of 220 h. Recently, Humphreys et al.^36^ demonstrated that it is possible to improve the entanglement rate by a couple of orders of magnitude, but this comes at the expense of the overall state fidelity and hence lower CHSH violations. While our protocol can yield positive asymptotic key rates in these noise regimes, a relevant question to consider is the number of rounds required to achieve security in a finite-key analysis.

To make this concrete, we analyse the finite-key security of our protocol using the proof technique from a recent work^37^ (see Supplementary Note 4 for the proof sketch). In particular, we compute the finite-key rates for both collective and general attacks, with the analysis of the latter making use of the entropy accumulation theorem^38–40^, which essentially certifies the same asymptotic rates as in the collective attacks scenario. (An alternative approach may be the quantum probability estimation technique^41^.) Our results are summarised in Fig. 3, focusing on the experiments from Hensen et al.^14^ and Rosenfeld et al.^17^ (which can achieve positive asymptotic key rate, as mentioned above). We see from the plots that they require ~10^8^ and 10^10^ measurement rounds, respectively to achieve positive finite-size key rates against general attacks. In these experimental implementations, this number of rounds is still currently out of reach (assuming realistic measurement time). Overall, however, given future improvements on these experimental parameters, our protocol would attain higher asymptotic rates than the original protocol^2^, and hence also require fewer rounds to achieve positive finite-key rates.

To further improve the robustness and key rates, there are a few possible directions to take. For one, we can consider the full input–output probability distribution instead of just taking the CHSH violation. Since the latter only uses part of the available information, more secrecy could potentially be certified by finding methods to compute secret key rates that take into account the full probability distribution estimated from the experiment. Such a method for general Bell scenarios was recently developed^42^; however, we found (see Supplementary Note 1) that the bounds it gives in this case are not tight, and are slightly worse than the results presented above. Another possible approach specialized for 2-input 2-output scenarios is presented in Tan et al.^37^, which is potentially more promising for such scenarios.

Box 1 Proposed device-independent quantum key distribution protocol **1. Measurements:** This step is carried out in *N* rounds, where *N* is assumed to be asymptotically large. In each measurement round, Alice’s and Bob’s inputs, denoted by *X* ∈ {0, 1} and *Y* ∈ {0, 1, 2, 3}, respectively, are drawn according to the following probability distributions: *P*(*X* = 0) = *p*, *P*(*X* = 1) = 1 − *p*, *P*(*Y* = 0) = *qp*, *P*(*Y* = 1) = *q*(1 − *p*) and *P*(*Y* = 2) = *P*(*Y* = 3) = (1 − *q*)/2, where 0 ≤ *p*, *q* ≤ 1. Once Alice and Bob enter their inputs into their respective devices, they each obtain a measurement outcome, which we denote by *A*_*X*_ ∈ {0, 1} and *B*_*Y*_ ∈ {0, 1}, respectively.

**2. Sifting:** Alice and Bob announce their measurement inputs over an authenticated public channel. This allows them to identify two common subsets of their measurement data: a pair of raw keys^32^ of size ~ *q*(*p*^2^ + (1 − *p*)^2^)*N* (corresponding to *Y* ∈ {0, 1} and *X* = *Y*) and a pair of parameter estimation data of size ~ (1 − *q*)*N* (corresponding to *Y* ∈ {2, 3}). Alice and Bob discard the remaining measurement data.

**3. Parameter estimation:** Alice and Bob publicly reveal their measurement outcomes from the parameter estimation data set and compute the underlying CHSH value:

$$S=\max \{2,{C}_{12}-{C}_{02}-{C}_{03}-{C}_{13}\},$$

where *C*_*XY*_ = *P*(_*AX*_ = *B*_*Y*_∣*X*, *Y*) − *P*(_*AX*_ ≠ *B*_*Y*_∣*X*, *Y*) is the correlation function of *X*, *Y*. Alice and Bob proceed to the next step if *S* > *S*_tol_, where *S*_tol_ is a predefined threshold value. Otherwise, they abort the protocol.

**4. One-way error correction and verification:** In the first part, Alice computes a syndrome based on her raw key (denoted by **L**) and sends it to Bob via the public channel, who then uses the syndrome and his raw key to recover Alice’s key. In the second part, they perform an error verification by comparing the hash values of their raw keys. Alice and Bob proceed to privacy amplification if the hash values are identical, otherwise they abort the protocol.

**5. Privacy amplification:** Alice and Bob perform privacy amplification to remove Eve’s information about Alice’s raw key. Once this is completed, Alice and Bob are left with a pair of identical secret keys.

## Methods

Here, we outline the main ideas of our security analysis. The core of the security analysis is a reliable lower-bound estimate on the conditional von Neumann entropy of Eve. The complete analysis is deferred to the accompanying Supplementary Note 1.

### Average secret key rate

Conditioned on the key generation rounds, Alice and Bob would pick their inputs (basis choices) according to a probability distribution (*p*, 1 − *p*). As discussed in the main text, this distribution acts as a free parameter in our protocol and has to be adapted to a given set of channel parameters (*S*, *Q*) in order to obtain an optimal performance. In the following we will, therefore, outline how the final key rate *K*_*∞*_ and secret fraction *r*_*∞*_ are given as functions of (*p*, *S*, *Q*).

The secret fraction in a round of the protocol, in which the measurements *A*_*X*_ and *B*_*Y*_ are obtained, can be computed using the Devetak-Winter bound ^43^ under the assumption of collective attacks,

5 $${r}_{\infty }^{{A}_{X}{B}_{Y}}\ge H({A}_{X}| E)-H({A}_{X}| {B}_{Y}).$$

Here the term *H*(*A*_*X*_∣*B*_*Y*_) only depends on the statistics of the measured data of Alice and Bob, and can therefore be directly estimated in an experiment. For binary measurements this quantity can be furthermore expressed by the respective bit error rate ${Q}_{{A}_{X}{B}_{Y}}$ via

6 $$H({A}_{X}| {B}_{Y})\le h({Q}_{{A}_{X}{B}_{Y}}).$$

In our protocol, a key generation round is obtained whenever Alice and Bob perform measurements *A*_*X*_ and *B*_*Y*_ with *X* = *Y*. The probability that Alice and Bob perform the measurements *A*_0_ and *B*_0_ is *p*^2^ and the probability that they perform *A*_1_ and *B*_1_ is (1−*p*)^2^. When the error correction is done for both cases, (*A*_0_, *B*_0_) or (*A*_1_, *B*_1_), separately, we obtain the overall asymptotic key rate as sum of the individual secret fractions weighted by their respective probability. This gives

7 $${K}_{\infty }\ge\; 	{p}^{2}{r}_{\infty }^{{A}_{0}{B}_{0}}+{(1-p)}^{2}{r}_{\infty }^{{A}_{1}{B}_{1}}\\ =\;	{p}^{2}H({A}_{0}| E)+{(1-p)}^{2}H({A}_{1}| E)\\ 	-{p}^{2}H({A}_{0}| {B}_{0})-{(1-p)}^{2}H({A}_{1}| {B}_{1})\\ \ge\;	{p}_{s}(\lambda H({A}_{0}| E)+(1-\lambda )H({A}_{1}| E)\\ 	-\lambda h({Q}_{{A}_{0}{B}_{0}})-(1-\lambda )h({Q}_{{A}_{1}{B}_{1}}))\\ :=\;	{p}_{s}{r}_{\infty },$$

where the success probability *p*_*s*_ and the relative distribution of the basis choices (*λ*, (1 − *λ*)) are given by

8 $${p}_{s}=\left({p}^{2}+{(1-p)}^{2}\right)=1-2p+2{p}^{2}$$

and

9 $$\lambda =\frac{{p}^{2}}{1-2p+2{p}^{2}},$$

respectively. As mentioned in the main text we also write

10 $$H(Z| E{{\Theta }}):=\lambda H({A}_{0}| E)+(1-\lambda )H({A}_{1}| E)$$

where Θ denotes a binary random variable (distributed by (*λ*, (1 − *λ*))) that (virtually) determines which basis pair, (*A*_0_, *B*_0_) or (*A*_1_, *B*_1_), is picked in a successful key generation round in order to generate the values of a combined random variable *Z* = *A*_Θ_.

### Device-independent entropic uncertainty relation

The only term in the key rate formula (7) that cannot be directly obtained from the measurement data is the conditional entropy *H*(*Z*∣*E*Θ). The main challenge here is thus to establish a reliable lower bound on this quantity assuming only the CHSH violation *S*. More specifically, we are interested in finding a function C^*^(*S*) such that

11 $$\ H(Z| E{{\Theta }})\ge {C}^{* }(S)$$

holds for all possible combinations of states and measurements (in any dimension) that are consistent with the observed CHSH value *S*. An inequality like equation (11) is commonly referred to as an entropic uncertainty relation, and in our case we are interested in relations with quantum side-information^33,44,45^. There is a vast amount of literature^34,46,47^ in which relations of this form^33,44,45,48^ or similar^49–55^ have been studied and several types of uncertainty relations have been discovered. A typical family of entropic uncertainty relations, which is close to our problem, is that proposed by Berta et al.^33^ and the weighted generalisation of it from Gao et al.^45^. These inequalities, however, are not device-independent and require the measurement characterisation of at least one party, which unfortunately is not possible in our setting.

To the best of our knowledge, the only known entropic uncertainty relations for uncharacterised measurements are given by Tomamichel et al.^56^ and Lim et al.^24^. There, the uncertainty of the measurement outcomes with side-information is lower bounded by the so-called overlap of the measurements^33^, which in turn is further bounded by a function of the CHSH violation. Although these relations are applicable to uncharacterised measurements, they appear fairly weak when applied to our DIQKD proposal, i.e. they do not provide any improvement in the secret key rate when compared to the original protocol.

**Fig. 4:**
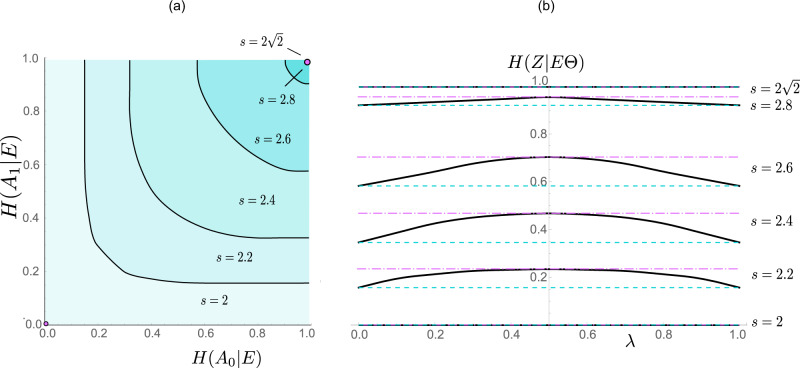
Device-independent uncertainty relations. In **a** the plot shows the device-independent uncertainty relation between *λH*(*A*_0_∣*E*) + (1 − *λ*)*H*(*A*_1_∣*E*), where the solid line is the fundamental uncertainty C*(*S*) for a given CHSH violation. The shaded region above the line hence represents the feasible region of (*H*(*A*_0_∣*E*), *H*(*A*_1_∣*E*)) given *S*. Evidently, when $S=2\sqrt{2}$, we see that *H*(*A*_0_∣*E*) = *H*(*A*_1_∣*E*) = 1 must be maximally random; indeed, $S=2\sqrt{2}$ corresponds to the case where Eve is completely uncorrelated with the devices and hence her best guess is limited to a random guess. In panel (b), the plot shows the minimal uncertainty C*(*S*) (the bottom dashed line) attained when *λ* = 0, 1 and one can see that C^*^(*S*) (solid line) for 0 < *λ* < 1 always gives a non-trivial advantage over the limiting case (like in the original DIQKD protocol).

The lower bound we establish in this work, i.e. the function C*(*S*), appears to be optimal in that it can be saturated by two-qubit states up to numerical precision (see Supplementary Note 1). C*(*S*) is depicted in Fig. 2b for *λ* = 0.5, and in Fig. 4b for continuous values of *λ* and $S\in \{2,2.2,2.4,2.6,2.8,2\sqrt{2}\}$. In Fig. 4a, we additionally plot the so-called uncertainty sets^47,54,57^ of our relation. These are sets that outline all the admissible pairs of entropies (*H*(*A*_0_∣*E*), *H*(*A*_1_∣*E*)) for a given lower bound on *S*. We also note that it may seem plausible to independently optimise each term *H*(*A*_0_∣*E*), *H*(*A*_1_∣*E*), instead of the sum of them. However, as shown in Fig. 4b, numerical results suggest that optimising the weighted sum of these terms is always better than optimising the individual terms: this also highlights where our DIQKD proposal improves over the original protocol.

### Computing C*(*S*)

As mentioned, the complete analysis of our lower bound on C*(*S*) is deferred to Supplementary Note 1. In the following, we will only outline the main steps of the analysis to reduce the computation of C*(*S*) to a sequence of problems that can be treated successively. The basic idea to find a way to compute C*(*S*) by only using known estimates (both analytical and numerical) that give a final lower bound that is reliable. This means that all the steps of the analysis assume the worst-case scenario, so that the final value is a strict lower bound on C*(*S*). In brief, the analysis uses a refined version of Pinsker’s inequality, semi-definite optimisation and an *ε*-net to achieve this goal.

Our first step is to reformulate the tripartite problem involving Alice, Bob and Eve to a bipartite one that involves only Alice and Bob. Following Tan et al.^42^, we note that conditional entropy terms like *H*(*A*_0_∣*E*) can always be reformulated as the entropy production *H*(*T*_*X*_(*ρ*_*AB*_)) − *H*(*ρ*_*AB*_) of the quantum channel *T*_*X*_ on the post measurement state on the Alice-Bob system, which is defined as the change of von Neumann entropy of the system that is subjected to the quantum channel *T*_*X*_. In our case, this channel *T*_*X*_ is a pinching channel, defined as:

12 $${T}_{X}[\rho ]:=({{{\Pi }}}_{0}^{{A}_{x}}\otimes {\bf{1}})\ \rho \ ({{{\Pi }}}_{0}^{{A}_{x}}\otimes {\bf{1}})+({{{\Pi }}}_{1}^{{A}_{x}}\otimes {\bf{1}})\ \rho \ ({{{\Pi }}}_{1}^{{A}_{x}}\otimes {\bf{1}}),$$

where ${{{\Pi }}}_{a}^{{A}_{x}}$ denotes the projector associated with Alice’s measurement setting *x* and outcome *a*. Clearly, the pinching channel satisfies ${T}_{X}={T}_{X}^{2}={T}_{X}^{* }$ and acts complementary to the map that models Alice’s measurement. With this, we can further rewrite the entropy production as

13 $$	\lambda H({A}_{0}| E)+(1-\lambda )H({A}_{1}| E)\\ 	=\lambda H({T}_{0}[{\rho }_{AB}])-\lambda H({\rho }_{AB})\\ 	\quad+(1-\lambda )H({T}_{1}[{\rho }_{AB}])-(1-\lambda )H({\rho }_{AB})\\ 	=\lambda D({\rho }_{AB}| | {T}_{0}[{\rho }_{AB}])+(1-\lambda )D({\rho }_{AB}| | {T}_{1}[{\rho }_{AB}]),$$

where *D*(*ρ*∣∣*σ*) is the quantum relative entropy of *ρ* with respect to *σ*.

Then, we follow a proof technique in the original work on DIQKD^2,3^ to reduce the underlying *ρ*_*AB*_ to a mixture of two-qubit states, where it is assumed that the mixing is due to Eve. That is, since each party (Alice and Bob) performs only two binary measurements, their local measurement devices can be described by only specifying two projectors (whose dimensions are unspecified). The corresponding local algebras, which are generated by two projectors, are well investigated mathematical objects^58^ for which a central theorem^59^ states that their representation can be decomposed into 2 × 2 (qubit) blocks and a commuting rest. Correspondingly, this allows us to conclude (details in the Supplementary Note 1) that the desired uncertainty bound can be decomposed accordingly as a convex combination:

14 $${C}^{* }(S)\ge 	\mathop{{\rm{inf}}}\limits_{\mu }\ \mathop{\int}\nolimits_{S^{\prime} = 2}^{2\sqrt{2}}\mu (dS^{\prime} )\ {C}_{{{\mathbb{C}}}^{4\times 4}}^{* }(S^{\prime} )\\ 	\;{\rm{s}}.{\rm{th}}.:\ \mu ([2,2\sqrt{2}])\le 1,\ \mu \ge 0\\ 	 \mathop{\int}\nolimits_{S^{\prime} = 2}^{2\sqrt{2}}\mu (dS^{\prime} )S^{\prime} =S,$$

where ${C}_{{{\mathbb{C}}}^{4\times 4}}^{* }(S^{\prime} )$ is a lower bound on the conditional entropy *H*(*Z*∣*E*Θ) for projective measurements on two qubits. Here, we note that once a bound on ${C}_{{{\mathbb{C}}}^{4\times 4}}^{* }(S^{\prime} )$ is established, the optimisation overall measures *μ*, which can be geometrically interpreted as taking a convex hull, is straightforward to perform. As shown in Fig. 5, the situation for the optimisation corresponding to ${C}_{{{\mathbb{C}}}^{4\times 4}}^{* }(S^{\prime} )$ can now, w.l.o.g., be fully described by specifying a two-qubit state and two angles (*φ*, *ω*) that describe the relative alignment of Alice’s and Bob’s measurements.

**Fig. 5:**
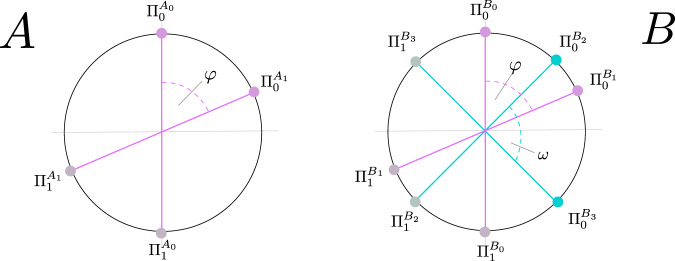
Measurement setting for two-qubit states. Alice has two projective measurement that are described by projectors $({{{\Pi }}}_{0}^{{A}_{0}},{{{\Pi }}}_{1}^{{A}_{0}})$ and $({{{\Pi }}}_{0}^{{A}_{1}},{{{\Pi }}}_{1}^{{A}_{1}})$ with relative angle *φ*. Bob has in total four measurements: for key generation, ideally he should perform measurements *B*_0_ and *B*_1_ which are aligned with Alice’s measurements *A*_0_ and *A*_1_ to minimise the quantum bit error rates. The security analysis only depends on Bob’s measurements *B*_2_ and *B*_3_, which are w.l.o.g. specified by a relative angle *ω*.

Although the problem has been reduced to two-qubit states and projective qubit measurements, a direct computation of ${C}_{{{\mathbb{C}}}^{4\times 4}}^{* }(S^{\prime} )$ is still an open problem as there are no known proof techniques that can be applied to our situation. To that end, we employ a refined version of Pinsker’s inequality (see Theorem 1 in the Supplementary Note 3),

15 $$D(\rho \| T[\rho ])\ge {\mathrm{log}}\,(2)-{h}_{2}\left(\frac{1}{2}-\frac{1}{2}\| \rho -T[\rho ]\|_{1}\right),$$

to obtain a lower bound on the relative entropy in (13) in terms of the trace norm.

The big advantage of establishing estimates in terms of the trace norm is that a minimisation thereof can be formulated as a semi-definite program (SDP). (In fact, it is possible in principle to use this inequality to bound the entropy without reducing the analysis to qubits, though the resulting bounds in that case do not appear to be very tight. We discuss this in detail in Supplementary Note 1.)

With that, the overall optimisation problem at hand now reads

16 $$\inf_{\varphi \in [0,\pi /2]}\, \Bigg\{ \inf_{{{\bf{b}} \atop {\| {\bf{b}}\| _{2} = 1}}}\, \Bigg[\begin{array}{ll} \inf_{\rho } &\lambda \delta (\rho ,0)+(1-\lambda )\delta (\rho,\varphi )\\ {\rm{s}}.{\rm{th}}.:&\left\langle{F}_{0}+{\bf{F}}\cdot {\bf{b}}\right\rangle_{\rho}=S\end{array}\Bigg]\Bigg\}$$

where

17 $$\delta \left(\rho ,\varphi \right):=\| \left\{\rho ,Q(\varphi )\right\}-2Q(\varphi )\rho Q(\varphi )\|_{1}$$

and constraints that are linear in *ρ* given by 4 × 4-matrices

18 $${F}_{0}\ {\rm{and}}\ {\bf{F}}\cdot {\bf{b}}={b}_{x}{F}_{x}(\varphi )+{b}_{z}{F}_{z}(\varphi ),$$

where *Q*(*φ*), *F*_*x*_(*φ*) and *F*_*y*_(*φ*) depend on *φ* in terms of the first and second order in $\cos (\varphi )$ and $\sin (\varphi )$. In the above expression, **b** is a vector on a (2-norm) unit sphere that arises from reformulating the description of Bob’s measurements.

This optimisation can be solved in three stages, indicated by boxes in (16):

(i) The optimisation within the square bracket [ ] over *ρ* is an SDP on 4 × 4 matrices, which can be efficiently solved^60^.
(ii) The optimisation in the curly bracket { } over **b** is performed by relaxing the continuous optimisation over the (2-norm) unit sphere to a discrete optimisation on a sequence of polygonial approximation (similar to the method used in Schwonnek et al.^61,62^). Also this optimisation can be performed with reliable lower bounds to the order of any target precision.
(iii) The last optimisation runs over the single parameter *φ* coming from a bounded domain. Hence, it is possible to efficiently tackle this optimisation by an *ε*-net. In order to do so it is required to provide an error estimate (for the magenta and the black box) for all *φ* that are located in an *ε*-interval around some *φ*_0_. Note that all previous optimisations are linear in *ρ* and **b** and only depend in the second order on $\cos (\varphi )$ and $\sin (\varphi )$ (which are bounded functions of *φ*).

## Supplementary information

Supplementary Information

## Data Availability

Data sharing is not applicable to this paper as no datasets were generated or analysed during the current study.

## Code availability

The code supporting the plots within this paper is available from the corresponding author upon reasonable request.
